# Does Male Skin Care Content on Instagram Neglect Skin Cancer Prevention?

**DOI:** 10.2196/50431

**Published:** 2024-03-13

**Authors:** Alexa Carboni, Olnita Martini, Jessica Kirk, Nathaniel A Marroquin, Corinne Ricci, Melissa Cheng, Mindy D Szeto, Kayd J Pulsipher, Robert P Dellavalle

**Affiliations:** 1 College of Osteopathic Medicine Rocky Vista University Greenwood Village, CO United States; 2 Western University of Health Sciences College of Osteopathic Medicine of the Pacific Pomona, CA United States; 3 Department of Dermatology University of Colorado Anschutz Medical Campus Aurora, CO United States; 4 Department of Dermatology Campbell University at Sampson Regional Medical Center Wilmington, NC United States; 5 Dermatology Service US Department of Veterans Affairs Rocky Mountain Regional Medical Center Aurora, CO United States; 6 Colorado School of Public Health Aurora, CO United States

**Keywords:** men, male, male skin care, male skincare, sunscreen, sun protection, photoprotection, anti-aging, skin cancer prevention, Instagram, social media, marketing, advertising, dermatology, dermatologist, skin, man, oncology, oncologist

## Abstract

This research letter assesses male skin care content on social media in order to bring to light the lack of content regarding skin cancer prevention posted on Instagram for male audiences.

## Introduction

Social media platforms can be efficient and engaging avenues for delivering information to target audiences [1]. A recent survey showed that 42% (n=1060) of US adults obtain health care information via social media, and 45% of respondents would take health-related actions after viewing medical content on these platforms [1]. Social media outreach regarding male skin care and sun protection may be an unrealized opportunity as an effective approach for skin cancer prevention, especially considering that men comprised most new skin cancer cases worldwide in 2020 (men: n=896,192, 59%; women: n=626,516, 41%; calculated based on data from Sung et al [2]), including cases of melanoma (men: n=173,844, 54%; women: n=150,791, 46%) and nonmelanoma (men: n=722,348, 60%; women: n=475,725, 40%) of the skin. Despite there being scientific evidence that consistent topical sunscreen use aids in the prevention of most skin cancers, the vast majority of men often neglect sunscreen compared to women, statistically [3]. Furthermore, male skin could also be more susceptible to UV damage, photoaging, and greater levels of UV exposure [4]. These patterns may be associated with a lack of tailored messaging from sources of health information [3]. Traditional advertising for male-focused skin care was mostly related to beard care, razors, and shaving products, and men historically were less likely to be receptive to targeted marketing content overall [5]. However, social media may have shifted attitudes such that influencer endorsements are now the most reliable form of outreach to both men and women [6].

## Methods

We aimed to evaluate male skin care social media on Instagram (Meta Platforms) and highlight any potential gaps in content related to sun safety and sunscreen use. Independent researchers investigated the following five relevant Instagram hashtags from January through March 2023: #maleskincare, #skincareformen, #skincaremen, #maleskincareroutine, and #maleskincareproducts. A total of 60 top posts were collected for each hashtag, after excluding posts with no likes, accounts with <20 followers, and videos. Posting dates, account names, followers, likes, and types of products advertised were recorded. A third reviewer categorized each post (N=300) by the topic or product discussed, as follows: beard/hair care, antiaging, cleansing, skin care routine, skin care educational infographics, acne, sunscreen, moisturizers, fragrance, or scar care.

## Results

Sunscreen comprised only 4.7% (14/300) of all topics or products promoted, while skin care routines were the most common (83/300, 27.7%; Table 1). The “skin care routine” category encompassed posts that focused on product lines or groups of products that could be used in a skin care routine, rather than centering on 1 product. Posts regarding beard/hair care (43/300, 14.3%), antiaging (45/300, 15%), cleansing (35/300, 11.7%), educational infographics about general skin care (31/300, 10.3%), acne (4/300, 1.3%), moisturizers (39/300, 13%), fragrance (1/300, 0.3%), and scar care (5/300, 1.7%) were also examined.

**Table 1:** Numbers and percentages of male skin care Instagram posts by topic.

Topic discussed	#maleskincare posts (N=60), n	#skincareformen posts (N=60), n	#skincaremen posts (N=60), n	#maleskincareroutine posts (N=60), n	#maleskincareproducts posts (N=60), n	Posts (N=300) by topic, n (%)
Beard/hair care	9	7	9	5	13	43 (14.3)
Antiaging	7	8	11	15	4	45 (15)
Cleansing	6	9	6	4	10	35 (11.7)
Skin care routine	19	13	19	18	14	83 (27.7)
Skin care educational infographic	7	14	2	1	7	31 (10.3)
Acne	1	1	1	1	0	4 (1.3)
Sunscreen	2	2	5	0	5	14 (4.7)
Moisturizers	9	6	6	11	7	39 (13)
Fragrance	0	0	1	0	0	1 (0.3)
Scar care	0	0	0	5	0	5 (1.7)

## Discussion

While the literature has suggested that men are motivated to use sunscreen due to prior knowledge of skin cancer risk reduction and a desire to appear younger [3], Instagram content related to sunscreen failed to address these factors. Shifting the focus of male skin care advertising may lead to greater interest in preventative measures and mitigate rising rates of skin cancer morbidity and mortality in men. Coupling sun protection and sunscreen promotion with the already substantial content on antiaging products may be promising, as sunscreen is known to have antiaging benefits. Interestingly, compared to women, men were more likely to rely on straightforward messaging and the credibility of the social media influencer when considering a product’s advantages and drawbacks [6]. Credentialed dermatologists therefore could play an important role in social media outreach and recommendations to men about sunscreen use, in conjunction with exploiting the more subtle marketing tactics that demonstrated prior success with male consumers [5]. This study underscores an opening for social media users and influencers to bring greater attention to an underrepresented issue.
